# Ultra–Minimally Invasive Medical Thoracoscopic Sympathectomy for Primary Palmar Hyperhidrosis

**DOI:** 10.1016/j.atssr.2025.12.024

**Published:** 2026-03-13

**Authors:** Zhengjun Li, Chang Liu, Tao Wang, Xiangchao Zhang, Guofeng Zhang, Sibo You, Yi Ren

**Affiliations:** 1 Department of Thoracic Surgery, Shenyang Chest Hospital, Shenyang, China; 2 Department of Anesthesia, Shenyang Chest Hospital, Shenyang, China

## Abstract

Endoscopic thoracic sympathectomy is an effective treatment of primary palmar hyperhidrosis. Currently, single-port thoracoscopy is the primary surgical approach. Ensuring efficacy and safety, reducing surgical obstacles, minimizing tissue damage, and enhancing cosmesis and aesthetics of the surgical incision are focal in clinical research on endoscopic thoracic sympathectomy. Herein, we report a simple and minimally invasive surgical technique that avoids collisions due to the telescope and endodissector crossing in single-port thoracoscopic surgery. This refers to the application of medical thoracoscopy combined with nonintubation anesthesia and perfusion index monitoring in the treatment of primary palmar hyperhidrosis.

Primary palmar hyperhidrosis (PPH) is functionally localized, with an unclear cause that is believed to be related to sympathetic nerve dysfunction. Patients with PPH often experience excessive sweating in the hands, feet, or armpits that seriously affects their lifestyle and may even cause both psychological and physical burdens. Endoscopic thoracic sympathectomy (ETS) is an effective treatment of PPH.^1^^,^^2^

With the development of minimally invasive techniques, ETS has evolved from 3-port and 2-port surgeries to more minimally invasive single-port surgeries,^3^^,^^4^ and anesthesia techniques have also continually improved. Ensuring efficacy and safety, reducing surgical obstacles, minimizing tissue damage, and enhancing cosmesis and aesthetics of the surgical incisions are focal in clinical research on ETS. Since the introduction of single-port thoracoscopy, it has been difficult to make further breakthroughs and progress in this technique. Herein, we report a simple, fast, minimally invasive, and minimally complicated surgical technique for emergency response: nonintubation with medical thoracoscopy to incise the thoracic sympathetic nerve for the treatment of PPH.

## Technique

**Figure 1:**
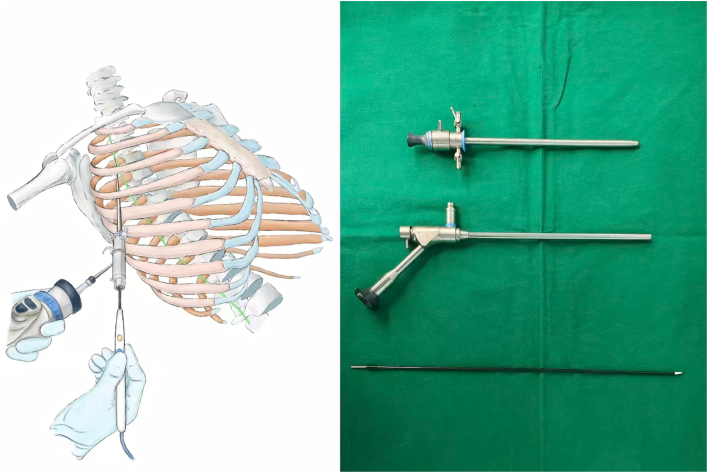
Internal medicine thoracoscopy.

**Figure 2:**
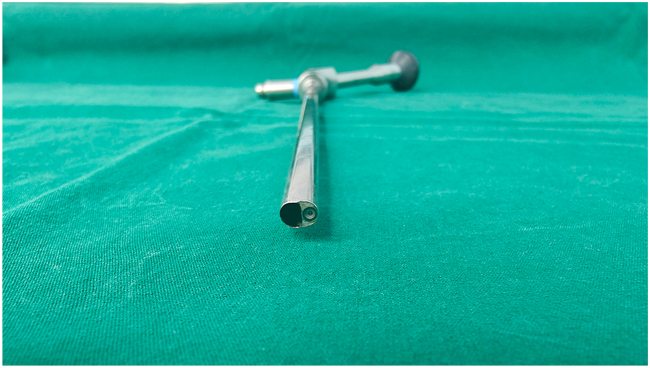
The operating channel of the internal medicine thoracoscope tube.

**Figure 3:**
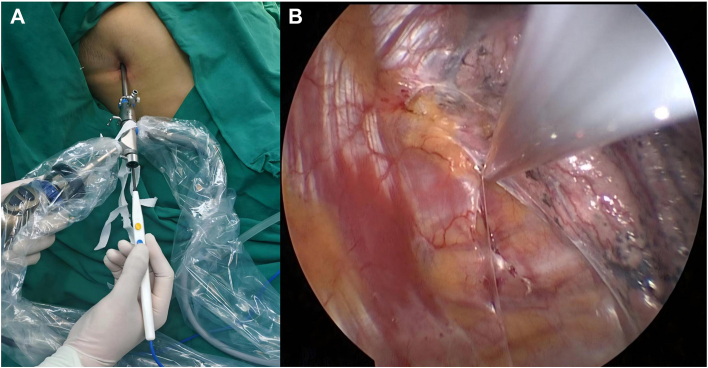
(A) Manual operation with both hands. (B) The fixed parallel field of vision of the electric hook and the lens.

Under general anesthesia, the patient was placed in a 30° to 45° semisitting position with both upper limbs abducted at 90° and fixed. Anesthesia was administered with a laryngeal mask or a single-lumen endotracheal tube. Right-sided surgery was performed first, followed by left-sided surgery. A skin incision of approximately 3 to 4 mm was made at the lateral edge of the pectoralis major and pectoralis minor muscles in the third or fourth intercostal space, as shown in Supplemental Figure 1. A blunt incision was made along the upper edge of the rib into the thoracic cavity by hemostatic forceps. A long trocar was inserted for medical thoracoscopy (Supplemental Figure 2) to create an artificial pneumothorax, and a medical thoracoscope was placed (Figure 1). The lung tissue was shielded to obtain a clear view, and the R3 and R4 sympathetic nerve segments were confirmed. An electrocoagulation knife was inserted into the thoracoscopy tube (Figure 2), and the R3 or R4 sympathetic nerve segments were transected parallel to the fixed field of view (Figure 3). The transected sympathetic nerve segment was used as the midpoint, and the parietal pleura and soft tissues were cauterized along the rib surface in the medial and lateral directions to destroy possible Kuntz bundles. Changes in pulse oximetry–derived perfusion index were observed during surgery to evaluate the surgical effect. After the absence of active bleeding in the surgical field was confirmed, the electrocoagulation hook was withdrawn, the pneumothorax machine was stopped, and an 18F red drainage tube was placed. The anesthesiologist inflated the lungs to expel gas from the pleural cavity (Supplemental Figure 3). The incision was sutured with vertical mattress sutures, and thoracic drainage was not performed. The Video shows the surgical procedure. The same method was used for left sympathetic nerve transection.

## Comment

PPH treatments include ETS, interventional therapy, and drug therapy. ETS remains the simplest and most convenient method of treating PPH. Surgeons have made many improvements in surgical anesthesia and techniques to meet patients' pursuit of cosmetic beauty and minimally invasive treatment. Anesthesia has evolved from double-lumen and single-lumen endotracheal intubation to nonintubation anesthesia. In terms of thoracoscopic surgical methods, the number of incisions has continually decreased, and single-port thoracoscopy is currently the primary method. Under single-port thoracoscopy, the modified microincision may simultaneously accommodate the trocar and electric hook, which may compress the surrounding soft tissues; the mutual influence of the instruments is also relatively obvious.^5^ The telescope and endodissector were inserted into the chest along the same axis. Congestion, crossing, and collisions during operations pose safety hazards. Moreover, removal of the puncture card affects the gas pressure inside the chest cavity, and bleeding from the incision also affects the surgery.

The diameter of the endoscopic thoracic tube for the medical thoracoscope (JS-1type; Shenyang Shen Da Endoscopy Co, Ltd) is 19.5F, and its length is 205 mm. The tube includes an optical lens and an operating channel. At present, medical thoracoscopy is mainly used for the diagnosis of pleural effusion in clinical practice, and it can be used for performing pleural or visceral pleural biopsies. However, it has not yet been applied in thoracic surgery. We performed the surgery we described using this kind of medical thoracoscope, which was connected to the Storz console and displayer.

Our method involves a skin incision of approximately 3 to 4 mm, blunt dissection into the thoracic cavity to minimize bleeding, and insertion of the trocar, followed by thoracoscopy. The procedure is simple. Therefore, it avoids the difficulty of inserting a cautery hook and the inconvenience caused by the simultaneous use of 2 instruments through a single incision in the conventional single-port ETS. It also overcomes the drawback of flexible endoscopy being unable to guide the operation direction.^6^ Even minor adhesions within the chest cavity can be managed, and the median unilateral operation time was approximately 5 minutes, significantly improving surgical efficiency. The parallel operation of the telescope and endodissector, with the operating field and endodissector remaining relatively fixed, enhanced the safety of the surgery.

We successfully and safely performed T3 or T4 thoracic sympathetic nerve resection in 19 patients using this surgical method, and all achieved good results. The control effect on perspiration from the hands is relatively good. For some patients, symptoms such as foot sweating, armpit sweating, or excessive sweating on the face have been partially alleviated. However, the effect is somewhat inferior compared with that of reducing sweating from the hands. Some surgeons have also performed the T3+T4 thoracic sympathetic nerve section surgery and have achieved certain results. However, there are also differences in terms of complications.

In conclusion, we successfully implemented this simple and efficient surgical method, nonintubated medical thoracoscopic sympathetic nerve dissection, for the treatment of PPH, which can be used as an alternative to conventional single-port thoracoscopic surgery.
