# Supplementary material for: Ultra–Minimally Invasive Medical Thoracoscopic Sympathectomy for Primary Palmar Hyperhidrosis
Source: Ann Thorac Surg Short Rep. 2026 Mar 13;4(2):733–6. doi: 10.1016/j.atssr.2025.12.024 (PMC13245509; doi:10.1016/j.atssr.2025.12.024)
Supplement: Supplementary Figures [file mmc1.docx]

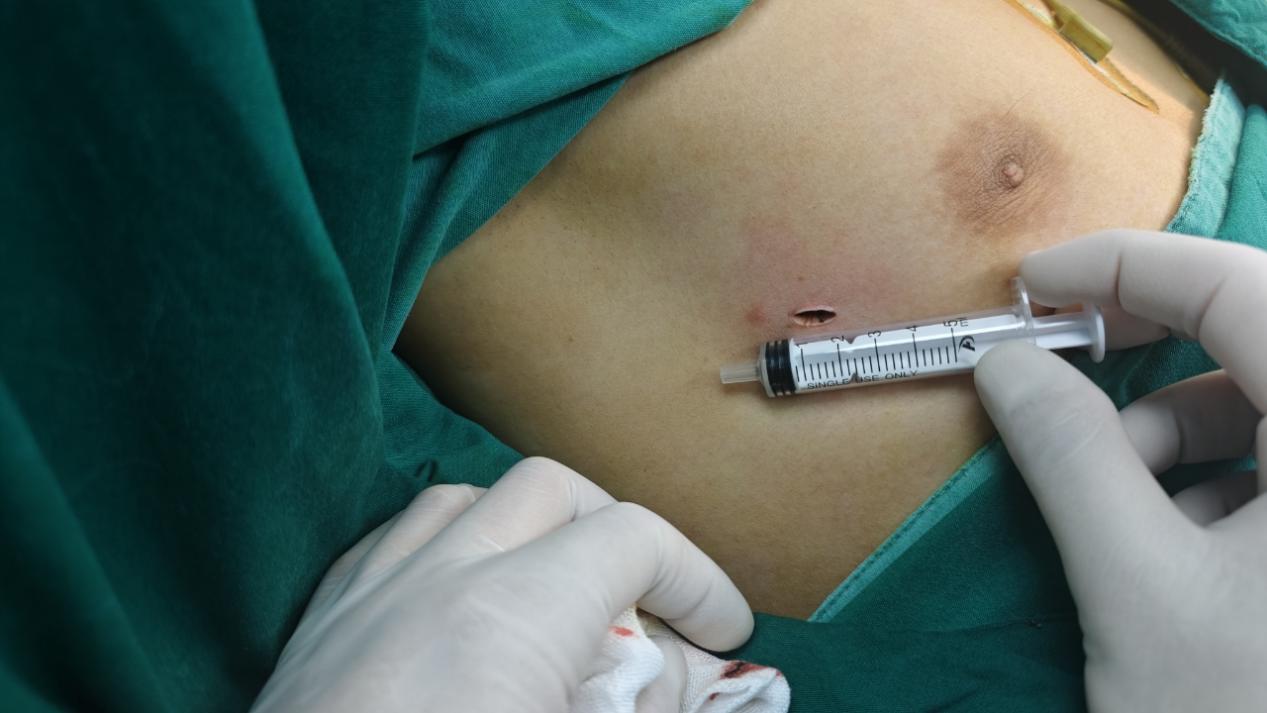


Figure S1. A 3-4 mm micro-incision at the third intercostal space.


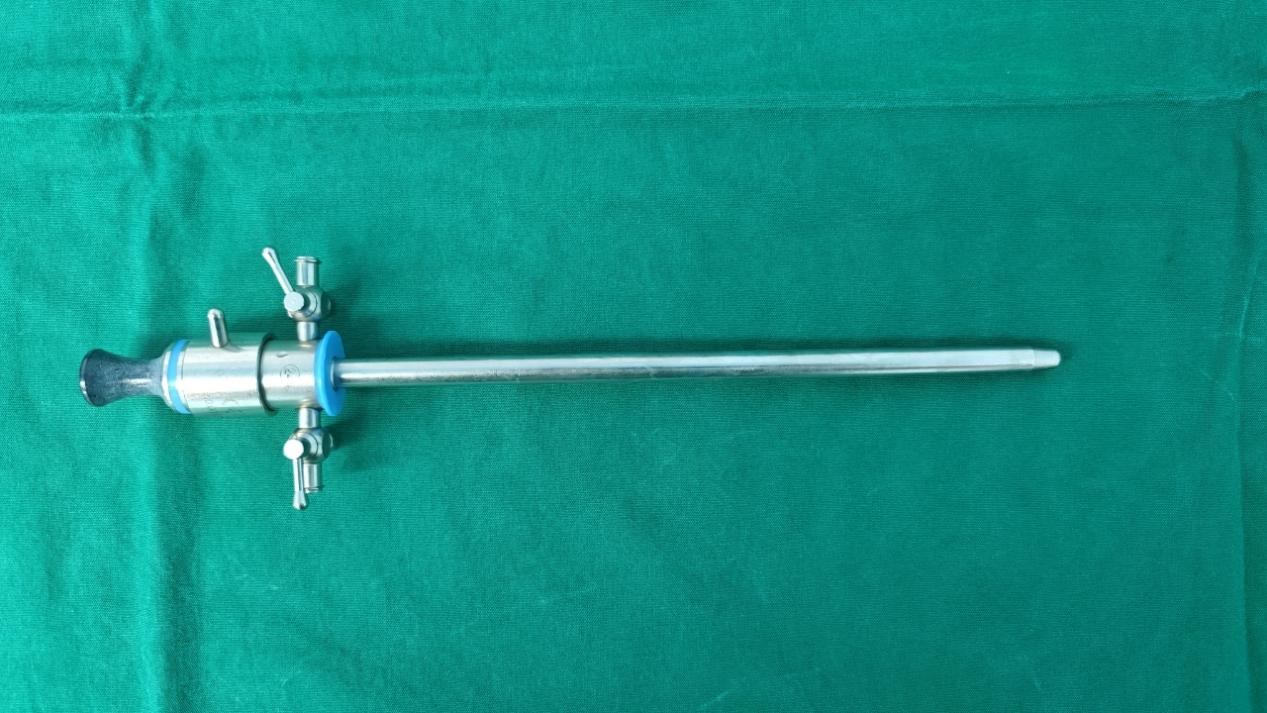


Figure S2. Long-tip card for internal medicine thoracoscopy.


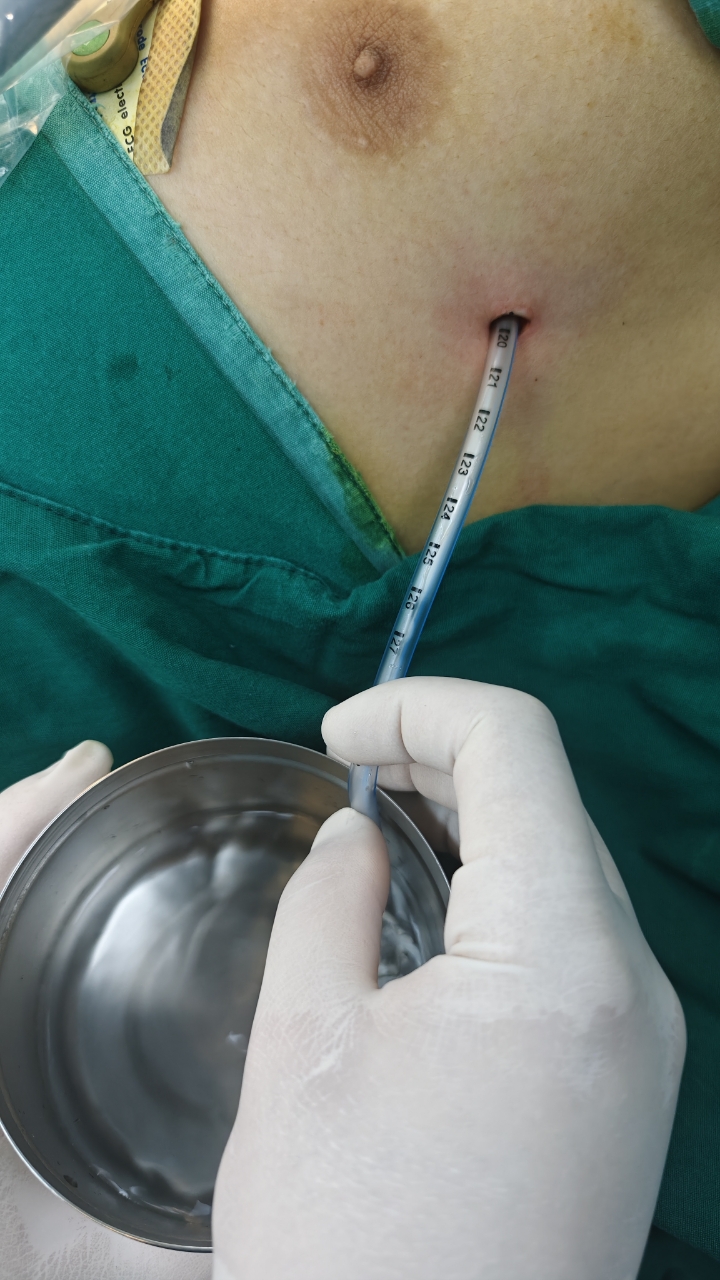


Figure S3. Expanding the lungs for air release.

**Figure legends**

Figure S1. A 3-4 mm micro-incision at the third intercostal space.

Figure S2. Long-tip card for internal medicine thoracoscopy.

Figure S3. Expanding the lungs for air release.
